# Effect of Tumor Targeted-Anthracycline Nanomedicine, HPMA Copolymer-Conjugated Pirarubicin (P-THP) against Gynecological Malignancies

**DOI:** 10.3390/jpm12050814

**Published:** 2022-05-18

**Authors:** Shintaro Yanazume, Jun Fang, Rayhanul Islam, Shanghui Gao, Hiroaki Kobayashi

**Affiliations:** 1 Department of Obstetrics & Gynecology, Faculty of Medicine, Kagoshima University, 8-35-1 Sakuragaoka, Kagoshima 890-8520, Japan; s-yana@m3.kufm.kagoshima-u.ac.jp; 2 Faculty of Pharmaceutical Sciences, Sojo University, Ikeda 4-22-1, Nishi-ku, Kumamoto 860-0082, Japan; fangjun@ph.sojo-u.ac.jp (J.F.); rayhanulislam88@gmail.com (R.I.); gaoshanghui94@gmail.com (S.G.); 3 School of Pharmacy, Queen’s University Belfast, 97 Lisburn Road, Belfast BT9 7BL, UK

**Keywords:** pirarubicin, P-THP, EPR effect, nanomedicine, drug delivery, ovarian cancer, uterine sarcoma

## Abstract

Anthracyclines are important for the treatment of gynecological malignancies, but their effects are modest, and one of the major reasons is the lack of a tumor-targeting property. To overcome this drawback, a poly (hydroxypropyl meta-acrylamide) conjugated with tetrahydropyraryl doxorubicin (P-THP) has been developed, which exhibits a highly tumor-specific accumulation owing to the enhanced permeability and retention effect. The effect of P-THP has been confirmed by using various cell lines and solid tumor models, while its effect on gynecological malignancies have not been investigated. In this regard, human uterine sarcoma cell line with metastatic potential MEA-SA C9 high, epithelial ovarian cancer cell line A2780 and its cisplatin-resistant line A2780cis, and DOX-resistant line A2780ADR were used in this study, and the therapeutic effect as well as the safety profiles of P-THP were investigated compared to native THP, cisplatin, and paclitaxel, which are commonly used for gynecological malignancies, both in vitro and in vivo. Similar to native THP, a dose-dependent toxicity of P-THP was identified in all cell lines. Moreover, the IC50 values in the 3 h following P-THP were 1.5–10 times higher than those at 72 h, though the intracellular uptake of P-THP in all cells were 2–10-fold less than THP. In vivo studies using xenograft tumor models revealed that P-THP significantly suppressed the MES-SA C9 high, A2780, and A2780cis tumor growth at the dose of 15 mg/kg (THP equivalent), which is three times above the maximal tolerance dose of native THP, while no body weight loss or acute death occurred. However, in A2780ADR cells and the xenograft model, no significant difference in the therapeutic effect was observed between THP and P-THP, suggesting that P-THP exhibits its effect depending on the release of the active free THP in tumor tissues, and thus the internalization into tumor cells. These findings indicates that P-THP has the potential as a therapeutic for gynecological malignancies to improve the therapeutic outcomes and survival rates of patients, even in refractory patients.

## 1. Introduction

Ovarian cancer is the second most common cause of death from gynecological cancer, and both advanced ovarian cancer and uterine malignant mesenchymal tumors have been commonly known to cause high mortality rates in gynecological malignancies [1,2]. Recent molecular-targeted drugs with PARP (poly (ADP-ribose/polymerase) inhibitors (PARPi), an angiogenic inhibitor, have improved survival rates in gynecological malignancies in ovarian cancer [3,4]. BRCA mutation with either tumors and/or germline, or homologous recombination deficiencies (HRD) in tumors, are good indications in patients to receive PARPi treatment, and the addition of an angiogenic inhibitor has proven to further prolong survival [4]. However, PARPi can be mainly used as maintenance therapy following the first-line platinum-containing treatment regimen because the cytotoxic efficacy is limited.

Cytotoxic chemotherapy is always the fundamental treatment for gynecological malignancies. Platinum-containing regimens were commonly used with paclitaxel plus carboplatin (TC) for first-line ovarian cancer treatment, and pegylated liposomal doxorubicin (Doxil^®^) plus carboplatin (PLD-C) is also used for recurrence [5]. The two regimens were directly compared in a randomized controlled trial, and the better therapeutic effect of Doxil was proven [5]. Progression-free survival (PFS) for the PLD-C was statistically superior to the TC arm (HR, 0.821; *p* = 0.005), where the median PFS was 11.3 months versus 9.4 months, respectively. Doxil^®^ is the first approved anticancer nanomedicine which exhibits prolonged circulation time and high tumor accumulation by taking advantage of the enhanced permeability and retention (EPR) effect [6]. Doxil^®^ has been approved for ovarian cancer as a promising therapeutic with a high therapeutic potential; however, clinical results could not indicate a sufficient anticancer activity to replace the existing cytotoxic agents such as TC [7], which is considered to be mostly due to the low release rate of its active pharmaceutical ingredients (API) [7]. Moreover, in a Japanese phase II trial of PLD-C in platinum-sensitive recurrent ovarian cancer, severe hematological toxicity of frequent grade 3–4 was observed to be associated with this treatment regimen, along with neutropenia (82%), thrombocytopenia (51%), anemia (17%), and nonhematological toxicity with palmar–plantar erythrodysesthesia (PPE) (45%) [8]. These findings suggest the necessity of the further elaboration of nanomedicines for clinical application.

Anthracycline-based chemotherapy, including doxorubicin (DOX) and pirarubicin (THP), has also been used for endometrial cancer, which causes a cytotoxic effect by DNA intercalation, topoisomerase activity inhibition, and the generation of reactive oxygen species. A recent clinical trial using DOX plus cisplatin (AP) revealed a similar outcome to the standard TC regimen [9]. Though many therapeutic regimens have been developed for gynecological malignancies, the treatment for patients resistant to conventional chemotherapy remains unestablished. In this context, molecular targeted therapy and precision medicine has gradually gained popularity in endometrial cancer treatment, and a recent trial for endometrial cancer patients using the molecular target drugs pembrolizumab plus lenvatinib has shown a significant survival benefit compared to conventional chemotherapy [10]. However, pembrolizumab monotherapy has shown less activity [11,12], and chemotherapy using cytotoxic drugs remains the first-line dominant treatment.

Anthracycline has been considered as the standard treatment choice for gynecological mesenchymal tumors with leiomyosarcoma or endometrial stromal sarcoma for more than 40 years. Since 2002, a combination of gemcitabine plus docetaxel was widely used as a first-line option for locally advanced or metastatic leiomyosarcoma [13]. However, a recent phase III clinical trial demonstrated no significant difference between this regimen and the single-agent DOX in patients, in which the patients which received six cycles of intravenous DOX of 75 mg/m^2^ exhibited the median progression-free survival of 23·3 weeks compared to the 23·7 weeks of the gemcitabine plus docetaxel regimen [14]. These results suggested the importance of anthracyclines for gynecological malignancies; therefore, the development of anthracycline-based drugs and regimens is of great necessity.

We have previously reported a case of completely cured refractory prostate cancer [15] by using a newly developed nanomedicine, poly (hydroxypropyl meta-acrylamide) (HPMA) conjugated with THP (P-THP) [16]. P-THP was developed at Maeda’s laboratory in 2014 as a polymer nanomedicine using a biocompatible HPMA copolymer [16]. The patient suffered from refractory advanced prostate cancer, having multiple metastatic lung nodules and multiple bone metastasis. Radiotherapy was targeted to the primary lesion, and P-THP was administered systemically for treating the metastatic lesions. P-THP doses ranged from 30 mg to 50 mg of free THP-equivalent dose/70 kg body weight every two to three weeks. The doses were administered seven times in five months and did not reveal any sign of toxicity, including cardiovascular failures. The patient has recorded no evidence of disease relapse for eight years [12].

P-THP has the apparent molecular size of about 40 kDa and a mean diameter of 8.2 mm, and highly tumor-specific accumulation owing to the EPR effect [16,17]. The antitumor activity of P-THP has been verified by using various cell lines and solid tumor models [16,17]; however, its effect on gynecological malignancies, including ovarian cancer and uterine mesenchymal tumors, has not been investigated. Particularly, the assessment of antitumor efficacy against the resistance of commonly used cytotoxic agents is important for applying clinical use. In this context, here we investigated the therapeutic effect of P-THP on uterine sarcoma and ovarian cancers, as compared with THP and the conventional chemotherapeutic drug paclitaxel. Furthermore, the applicability of P-THP for DOX- or cisplatin-resistant ovarian cancers, which frequently occur in clinic after chemotherapy using DOX or cisplatin, is also investigated, by which the insight into the potential of treatment strategies using P-THP for the gynecological malignancies are discussed.

## 2. Materials and Methods

### 2.1. Materials

Human uterine sarcoma cell line MES-SA, the GPF-transfected cell line (MEA-SA C9), and its selected subset with a high metastatic potential (MEA-SA C9 high) [15] were kindly provided by Prof. Yoshio Yoshida in the Department of Biochemistry, Medical Sciences, Fukui University, Japan. Epithelial ovarian cell lines A2780 (93112519) [16], and its cisplatin-resistant cell line A2780/cis (93112517) [17] and DOX-resistant cell line A2780/ADR (93112520) [16] were purchased from the European Collection of Authenticated Cell Cultures (ECACC) cell bank. THP, cisplatin, and paclitaxel were purchased from Wako Pure Chemical (Osaka, Japan). Doxil^®^ was purchased from Janssen Pharmaceutical K.K. (Tokyo, Japan). P-THP was synthesized as reported previously [13].

### 2.2. In Vitro Cytotoxicity Assay

MES-SA cells were cultured in McCoy’s 5A medium supplemented with 10% fetal bovine serum (FBS) and 2 μg/mL gentamicin solution under the 5% CO_2_/air at 37 °C. An amount of 1 μg/mL puromycin dihydrochloride was added to the MEA-SA C9 and MEA-SA C9 high cells during culture. A2780 cells were cultured in RPMI1640 with 10% FBS. An amount of 0.1 µM cisplatin was added to A2780/cis cells, and 0.1 µM DOX hydrochloride were added to A2780/ADR cells, respectively.

MTT assay was carried out for evaluating the cytotoxicity. The cells were seeded in a 96-well plate at 3000 cells/well. THP and P-THP of different concentrations were added to the cells, and the cells were further cultured for 3 h or 72 h. The medium was then removed, and fresh medium was added after the cells were washed three times with PBS. MTT assay was then performed. For cells treated for 3 h, further culture of 72 h was carried out before MTT assay.

### 2.3. Intracellular Uptake Analysis of THP and P-THP

Above-described cells of 5 × 10^5^ cells/well cells were seeded in 12-well plates and treated with 100 μg/mL (THP equivalent) of P-THP or THP for the indicated time periods. Then, the cells were washed twice with PBS, resuspended in 500 μL PBS, followed by sonication for 30 s (UR-21P^®^ (TOMY Co. Ltd., Tokyo, Japan)).

For detecting the released THP from P-THP, an equal volume of 0.2 M sodium bicarbonate buffer (pH 9.8) was added to the cell lysate and mixed, to which 600 μL of chloroform was added and mixed vigorously. After centrifugation (8000 rpm, 5 min), 400 μL of the lower chloroform layer (containing free THP) was collected. The amount of THP was then measured by using a fluorescence spectrometer (Infinite^®^ M200 (Tecan Trading AG, Männedorf, Switzerland); excitation wavelength, 488 nm, emission wavelength, 550–650 nm).

For measuring the total amount of THP including both P-THP and released free THP, equal volumes of 2 M HCl were added to the cultured cell lysate and treated at 50 °C for 1 h, followed by the same protocol as described above.

In a separate experiment, intracellular images derived from THP and P-THP were observed by the fluorescence microscope (BX50-33FLA2 camera DP74 (Olympus Co, Tokyo, Japan); excitation wavelength of 530–550 nm, fluorescent wavelength of 580 or higher nm).

### 2.4. In Vivo Antitumor Effect of P-THP

In vivo antitumor effect of P-THP was investigated by using xenograft models in mice, as compared with commonly used chemotherapeutic drugs for gynecological malignancies, i.e., THP, cisplatin, and paclitaxel. BALB/c nude mice in vivo were purchased from SLC, Shizuoka, Japan. The animals were maintained at 22 ± 1 °C and 55 ± 5% relative humidity with a 12 h light and dark cycle. All experiments were approved by the Animal Ethics Committees of Sojo University and performed according to the Laboratory Protocol for Animal Handling of Sojo University.

Tumor cells (1 × 10^7^) were implanted in the dorsal skin of BALB/c nude mice, and after 4–6 weeks when the tumors grew to 6–8 mm in diameter, the treatment was carried out. THP (5 mg/kg) and P-THP (15 mg/kg) were administered intravenously (i.v.) weekly for 3 times; CDDP (5 mg/kg) and paclitaxel (3 mg/kg) were administered i.v. weekly for the total of 4 injections.

The tumor volume (mm^3^), which was calculated as (W^2^ × L)/2 by measuring the width (W) and length (L) of the tumor, and body weight of the mice were recorded every 2–3 days during the period of the experiment. When the tumor reached the size of 4000 mm^3^, the mice were euthanized.

## 3. Results

### 3.1. Cytotoxicity Assay for Gynecological Malignancies of P-THP In Vitro

**Figure 1:**
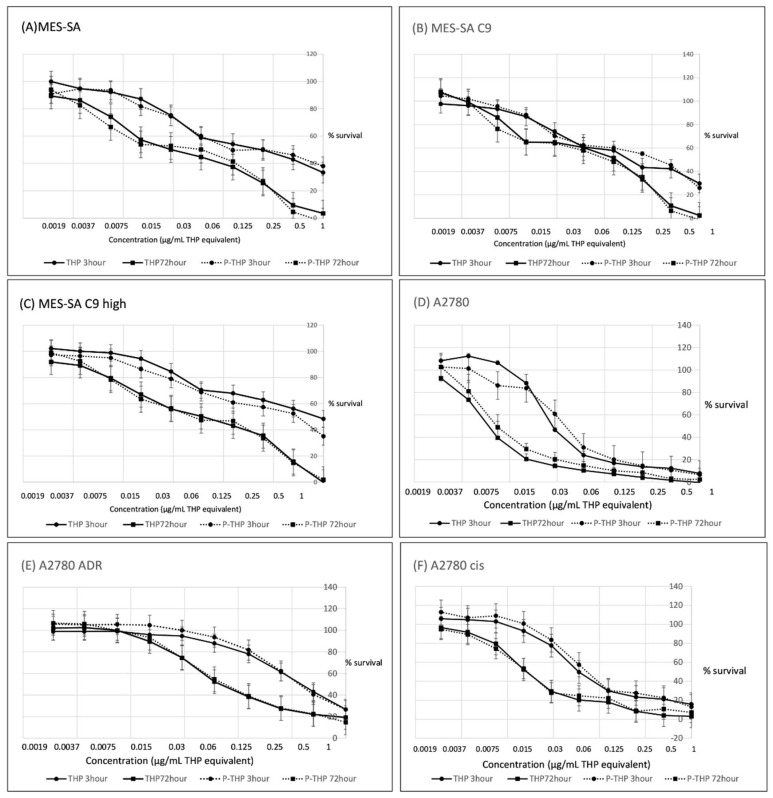
Cytotoxicity of THP or PTH for MES-SA (**A**), MES-SA C9 (**B**), MES-SA C9 high (**C**), A2780 (**D**), A2780/ADR (**E**), and A2780/cis (**F**) were assessed. Dose-dependent toxicity of P-THP, similarly to THP, were identified at both 3 h and 72 h incubation. Data are expressed as a mean percentage of control growth ± standard deviation (SD) (*n* = 8 replicates per concentration).

As seen in Figure 1A–F, in all tested cells, P-THP showed a dose-dependent cytotoxicity which is similar to free THP. The 50% inhibitory concentration (IC_50_) values of THP and P-THP against the different cell lines are indicated in Table 1. Moreover, time-dependency was also found for P-THP as well as free THP, in which the IC_50_, after 3 h of treatment, were 1.5–10 times higher than that after 72 h treatment (Figure 1). More importantly, after 3 h treatment, the IC_50_ values of P-THP were 2–5-fold higher than the free THP, whereas after 72 h treatment, the cytotoxicity of P-THP was almost similar to that of free THP (Table 1).

### 3.2. Intracellular Uptake of THP and P-THP

**Figure 2:**
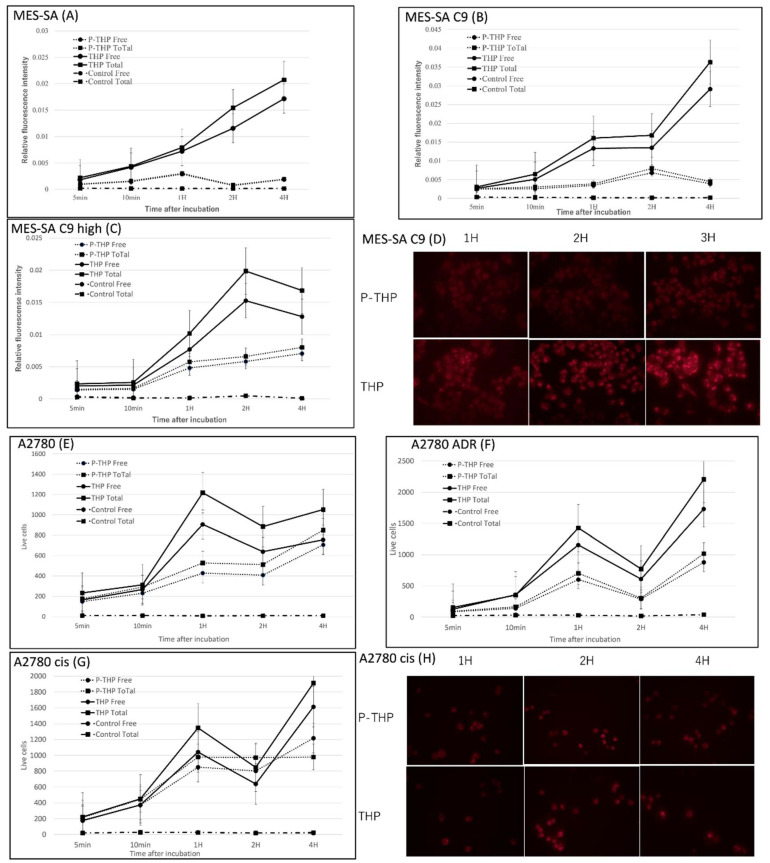
Intracellular uptake of THP and PTHP for MES-SA (**A**), MEA-SA C9 (**B**), MEA-SA C9 high (**C**), A2780 (**E**), A2780/ADR (**F**), A2780cis (**G**). The fluorescence imaging of MES-SA C9 (**D**) and A2780 cis (**H**) were also shown. ‘Free’ means released THP and ‘Total’ means total THP. Data are expressed as a mean percentage of control growth ± Standard Deviation (SD).

In Figure 2A–C, we firstly analyzed the intracellular uptake of THP and P-THP in MES-SA, MEA-SA C9, and MEA-SA C9 high cells. A time-dependent increase of internalization was observed (Figure 2A–C), which was also visualized by using fluorescence microscopy (Figure 2D). Moreover, the intracellular uptake of P-THP in all of the test cells were 2–10-fold less than THP, regardless of whether free THP or total THP (including contact P-THP) was measured. These findings suggested that the release of free THP is necessary for P-THP to exert is effect.

We then investigated the intracellular uptake of P-THP in ovarian cancer A2780 cells, its resistant cell line A2780/cis, and A2780/ADR, as shown in Figure 2E–H. Similarly, the uptake of THP was much better than that of P-THP in all cells, and the uptake of P-THP in ADR-resistant cell lines was also reduced, which was similar to THP. These findings further supported that the release of free THP, and the internalization into the cells, are critical steps for P-THP’s anticancer activity.

### 3.3. In Vivo Therapeutic Effects of P-THP against Gynecological Malignancies

**Figure 3:**
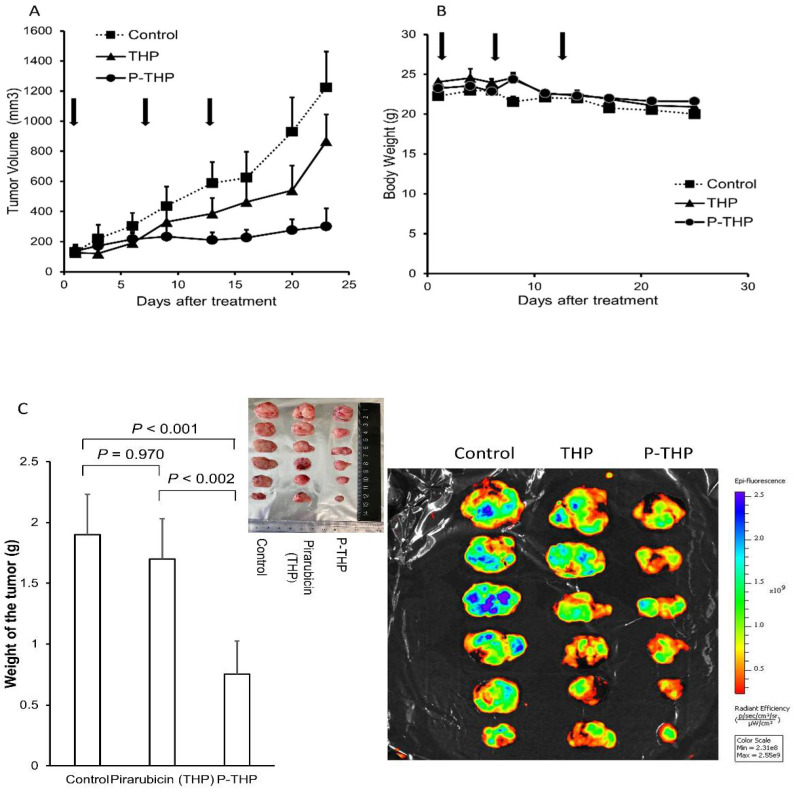

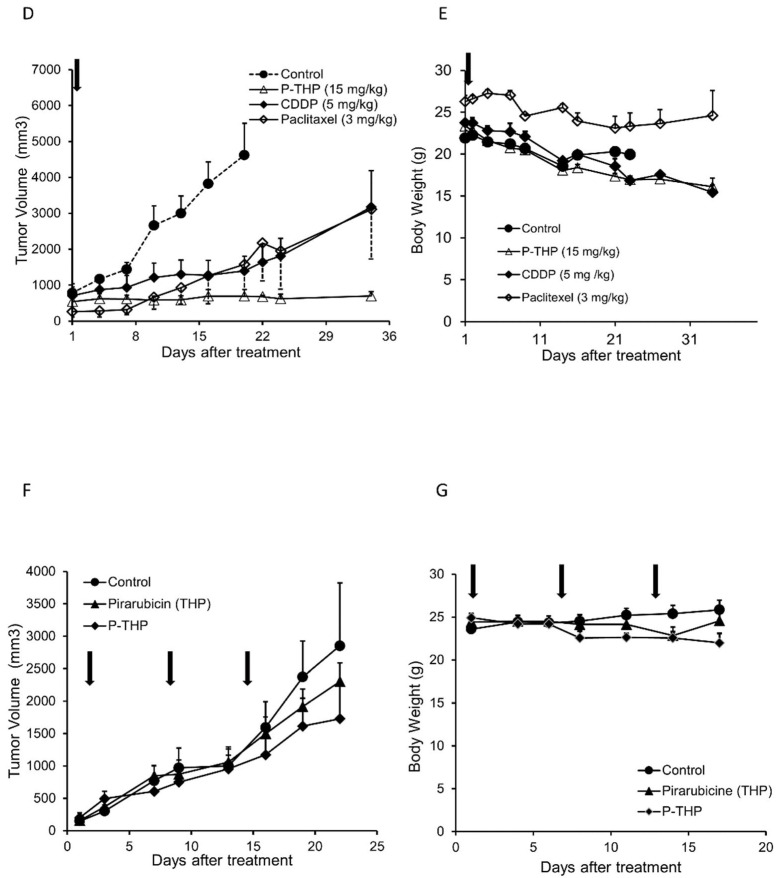
Antitumor effect of P-THP in vivo. MES-SA C9 high tumor-bearing mice (**A**,**B**): Free THP and P-THP at 5 mg/kg and 15 mg/kg of THP-equivalent doses are weekly administered three times from the tail vein when the tumor diameter is 6–12 mm. (**A**) Tumor volume (mm^3^) and (**B**) body weight (g) change. Data ± SEM (*n* = 4). MES-SA C9 high tumor-bearing mice (**C**): (**C**) Tumor weight after 36 days of treatment started of sacrifice mice revealed P-THP was significantly less than THP, as well as fluorescence imaging. Fluorescence images showed mice tumors treated with THP or P-THP. The tumors were cut in the middle of tumor nodules, and the cross-sectional views were shown. Data ± SEM (*n* = 4). A2780cis tumor model (**D**,**E**) of 1.5 × 10^7^ cells per inoculation were injected: THP and P-THP at 5 mg/kg and 15 mg/kg of THP-equivalent doses were weekly administered from 6–12 mm of initial tumor size (*n* = 3). Data are expressed as Data ± SEM. Paclitaxel (3 mg/kg) was used with 4 weekly i.v. injections. A2780/ADR tumor model (**F**,**G**) of 1.5 × 10^7^ cells per inoculation: THP and P-THP at 5 mg/kg and 15 mg/kg of THP equivalent doses were weekly administered from 4–10 mm of initial tumor size (*n* = 4). Data ± SEM (*n* = 4). Regarding the dosing of P-THP, 3 injections were carried out only in the A2780/ADR tumor model; for the other tumor model, one injection of P-THP was performed.

In the MES-SA C9 high tumor xenograft model, which is a highly aggressive tumor with high metastasis potential, P-THP significantly suppressed the tumor growth, which was much better than the effect of THP (Figure 3A), while no apparent body weight loss or acute death occurred (Figure 3B). The tumor weight of the P-THP treatment group at 36 days after treatment was significantly lower than both the control (*p* < 0.001) and THP treatment group (*p* < 0.002), whereas no significant decrease of tumor weight was found in the THP treatment group compared to the control group (Figure 3C,D).

More importantly, in the cisplatin-resistant A2780/cis xenograft model, compared to the anticancer drugs commonly used for epithelial ovarian tumors, i.e., cisplatin and paclitaxel, P-THP showed a clearly much improved antitumor effect, in which the tumor growth was almost completely inhibited up to 35 days after treatment (Figure 3D). Moreover, during the treatment, no apparent body weight loss was observed (Figure 3E).

However, in the DOX-resistant A2780/ADR tumor xenograft model, no apparent therapeutic effect was observed for P-THP, which was similar to free THP (Figure 3F,G).

## 4. Discussion

The anticancer activity of P-THP has been reported in various types of tumor cell lines, including cervical cancer (HeLa cells), melanoma (B16-F10), colon cancer (HCT116/C26), glioblastoma (U87-MG), and pancreatic cancer (SUIT2) cell lines [13,18,19]. All our cell lines in vitro were inhibited by P-THP in a concentration and time-dependent manner, which, 3 h after treatment, the cytotoxicity of P-THP was lower to that of the free THP (Table 1). These findings are similar with previous studies which indicated P-THP is less cytotoxic than THP. This is mostly attributed to P-THP’s slower uptake into the cell than P-THP, as evidenced in Figure 2. Similar phenomena were also seen in many other macromolecular agents [13], in which hydrophilic polymer components impede the internalization of drugs, known as the PEG dilemma [13,20,21]. However, after a long time treatment (i.e., 72 h), P-THP showed almost similar cytotoxicity as free THP (Table 1). We considered that it was the consequence of the release of free THP from P-THP. Because THP was conjugated to the HPMA polymer by the hydrazone bond, the release of free THP from P-THP occurred constantly and slowly in physiological pH, but rapid release was triggered at an acidic pH [13], so a long time incubation results in an increased amount of released free THP, and the decreased pH in the medium due to the tumor cell metabolism induced the release of more free THP, consequently resulting in a much-increased cytotoxicity of P-THP that is comparable to free THP. Further investigations are warranted to clarify this issue.

Regarding the in vivo antitumor activity of P-THP, we adopted the dosage of 5 mg/kg for THP and 15 mg/kg (THP equivalent) for P-THP, because 15 mg/kg is almost the LD_50_ of free THP, of which 40% of the tumor-bearing mice died at this dose in our previous study [13], while the LD_50_ of the P-THP was reported to be higher than 60 mg/kg [18], and no apparent side effects were found at 15 mg/kg [13]. In the implanted uterine sarcoma mice model, we clearly found that P-THP almost completely suppressed the tumor growth, in which the effect was much superior to the free THP, whereas no remarkable body weight loss was observed (Figure 3A, B). Regarding uterine sarcoma, anthracycline is still the first-line treatment for advanced uterine sarcoma. In a recent clinical trial [14] comparing gemcitabine and docetaxel versus DOX as first-line treatment, the survival outcomes were similar in both groups, while complications tended to be less with DOX. However, regarding THP, the response rate of single-agent THP was reported to be only 25%, which is mostly due to its high toxicities, i.e., febrile neutropenia (20%), fatigue (6%), and mucositis (14%), that limit the application and dosage of THP [19]. Development of new drugs is thus necessary to find potentially more effective treatments for advanced uterine sarcoma, and our current study strongly suggested that P-THP may become a candidate for uterine sarcoma.

Advanced ovarian cancer is highly chemo-sensitive, with over 70% of patients responding to first-line chemotherapy, but many cases finally succumb to chemo-resistant disease [20]. Cisplatin-resistant ovarian cancer patients were generally treated with single-agent liposomal DOX, or weekly paclitaxel and topotecan; however, the efficacy was low, at a 12–25% response rate [21,22]. In this study, in the cisplatin-resistant A2780/cis tumor model, we found that P-THP was strongly effective and exhibited a much better anticancer effect than not only cisplatin, but also paclitaxel (Figure 3D). However, by the present treatment protocol, no advantage was observed for P-THP than free THP in the DOX-resistant A2780/ADR tumor model (Figure 3F), as both P-THP and THP did not show a significant antitumor effect. These findings further supported the notion that P-THP exerts its effect by releasing free THP in the tumor tissue, in which the effect was similarly affected by the overexpressed outflow pump in A2780/ADR cells. However, this hurdle could be overcome by increasing the dosing of drugs, which the high tumor accumulation, and thus low toxicity, of P-THP will enable in dose escalation, which needs further investigations.

Since solid tumors have innumerable subpopulations (mutant cells, gene polymorphisms) even within the same patient, the surviving resistant clones may start to grow immediately if they are not eradicated by the administered antitumor agent. To achieve this goal, administering a high concentration of a drug that would kill all cancer cells, including resistant clones, is commonly considered. However, in clinical practice, high-dose administration is usually not possible due to the serious toxicity/side effects. In this regard, a drug delivery system that delivers drugs selectively to tumors is a useful and effective strategy, namely, tumor targeting. EPR effect-based nanomedicine is a well-recognized strategy of tumor targeting. The EPR effect was first demonstrated by Matsumura and Maeda in 1986 [23], which is a unique phenomenon in most solid tumors due to the abnormal anatomical and pathophysiological features of solid tumors, such as active angiogenesis, the large gap between endothelial cells of tumor blood vessels, the active production of various vascular permeability factors, and the lack of lymphatic drainage [23,24]. Based on the EPR effect, many nanomedicines have been developed, including polymer conjugates, polymeric micelles, liposome, nanoparticles, antibody drug conjugate, and so on, some of which are approved for clinical use, and more are in preclinical trials [25]. For example, recently squalene that is a triterpene in the cholesterol biosynthesis pathway widely distributed in nature, has been successfully developed as a nanoplatform for various therapeutic molecules and nucleoside analogues with gemcitabine, as well as doxorubicin and paclitaxel, and the the squalenoylated doxorubicin nanoassemblies exhibited much improved anticancer therapeutic efficacy with decreased cytotoxicity [26,27].

To maximize the effectiveness of nanomedicines, the following three conditions are considered necessary for a successful nanomedicine: (1) prolonged circulation time/stability in circulation and selective accumulation in the tumor by the EPR effect; (2) the rapid release of API responding to tumor environment; (3) active cellular uptake of the API into the tumor cell. P-THP is just such a sample designed and developed by these three criteria. Namely, P-THP has the potential to be an intelligent nanomedicine for the treatment of malignant tumors with a low toxicity by its following characteristics: (1) the selective accumulation in the tumor tissue, which is about 30 times higher than normal tissue due to the EPR effect [16]; (2) the hydrazone bond in P-THP as the acidic tumor environment-responsive linker, resulting in rapid low pH environment of the tumor tissue (5.5 to 6.5 compared to 7.4 in normal tissue) [16]; (3) the rapid intracellular uptake of free THP that is 30 to 50 times faster than DOX [28], which is contributed to, at least partly, the active transport systems, such as the glucose transporter in tumor cells that is 10 times higher than in normal cells.

In contrast, nanomedicines that could not satisfy the above criteria may suffer from an insufficient effect. For example, the block copolymer micelle-containing DOX (NK911, Nippon Kayaku Co., Ltd.; Tokyo, Japan) that encapsulates DOX via the noncovalent bond showed low stability in the blood stream, which releases 50% of the APIs within two hours, resulting in a relatively short plasma half-life (t_1/2_); consequently, it could not behave as a nanomedicine completely, thus exhibiting an insufficient therapeutic effect [29]. On the contrary, Doxil^®^ shows very high stability, having a very low rate of DOX release from the liposome, though it does exhibit high EPR effect-based tumor accumulation, and thus the therapeutic outcome is not fully satisfied [6,30].

Another important potential of P-THP, besides its superior antitumor effect, is its low toxicity/high safety. THP itself has 38% less cardiotoxicity than DOX, and its maximum cumulative-tolerated dose (MCTD) is 650 mg/m^2^ [31]. By taking advantage of the EPR-based tumor selective accumulation, P-THP’s toxicity is much less than THP, as evidenced by many previous studies using solid tumor models [16,28]. The present study also clearly suggested the high safety of P-THP in all tumor models, as the P-THP treatment did not induce any apparent loss of body weight (Figure 3B,E,G). Future study will focus on the determination of the maximum cumulative-tolerated dose (MTD) of P-THP, the acceptable safety level and safety profile of P-THP, and the safety and efficacy of P-THP monotherapy or combination therapy in recurrent or newly diagnosed disease, such as leiomyosarcoma, for the clinical development of P-THP.

In conclusion, the history of gynecologic oncology has proven that multidisciplinary treatment over the tumor heterogeneity and drug resistance is required for eradicating the malignancies. Continuing development of various types of antitumor agents such as immunotherapies, precision medicine, radiotherapy, and cytotoxic agents are warranted. The present study shows that P-THP, utilizing the EPR effect, may improve survival rates without major complications, even in refractory patients. With the potential of tumor targeting and nontoxic properties, we anticipate the application of P-THP for gynecological malignancies. Further extensive studies of P-THP are necessary and warranted.

## Figures and Tables

**Table 1:** In vitro cytotoxicity (IC_50_) values of THP and P-THP in A2780, A2780cis, and A2780ADR after 3 h and 72 h of incubation at 37 °C.

	Incubation Times	LC50 (μg/mL)		Incubation Times	LC50 (μg/mL)
**THP**			**P-THP**		
MES-SA	3 h	0.063	MES-SA	3 h	0.253
	72 h	0.017		72 h	0.042
MES-SA C9	3 h	0.207	MES-SA C9	3 h	0.251
	72 h	0.091		72 h	0.045
MES-SA C9 high	3 h	0.029	MES-SA C9 high	3 h	0.378
	72 h	0.026		72 h	0.051
A2780	3 h	0.111	A2780	3 h	0.065
	72 h	0.018		72 h	0.044
A2780cis	3 h	0.050	A2780cis	3 h	0.111
	72 h	0.068		72 h	0.028
A2780ADR	3 h	0.904	A2780ADR	3 h	0.567
	72 h	0.192		72 h	0.189

## Data Availability

The data presented in this study are available on request from the corresponding author.
